# Comparable disease course in pediatric celiac disease with or without endoscopic confirmation at high anti-TG2-IgA-antibody titers

**DOI:** 10.3389/fnut.2026.1930734

**Published:** 2026-09-10

**Authors:** Sebastian Stricker, Lisa Marie Schönfeld, Frank Rommel, Pauline Grunst, Jan De Laffolie, Silvia Rudloff

**Affiliations:** 1 Department of General Pediatrics and Neonatology, Justus Liebig University Giessen, Giessen, Germany; 2 Department of General Pediatrics, University Children’s Hospital Marburg, University of Marburg, Marburg, Germany; 3 Department of Nutritional Science, Justus Liebig University Giessen, Giessen, Germany

**Keywords:** anti-TG2-IgA-antibodies, celiac disease, non-biopsy approach, pediatric gastroenterology, serological remission

## Abstract

**Background:**

The guidelines from the European Society for Pediatric Gastroenterology, Hepatology and Nutrition (ESPGHAN) enable the diagnosis of celiac disease (CD) omitting esophagogastroduodenoscopy (EGD) in patients with high anti-Transglutaminase 2 (TG2)-IgA-antibodies. However, there is uncertainty whether this approach is safe in terms of disease outcomes for all patients. Here, we aimed to investigate the course of anti-TG2-IgA-antibodies, symptomatic response, anthropometric measurements and routine laboratory values in CD patients presenting with high anti-TG2-IgA-antibodies diagnosed with or without EGD.

**Methods:**

We conducted a retrospective case-control study in two pediatric gastroenterology centers in Giessen and Marburg. CD patients presenting with high anti-TG2-IgA-antibodies (≥10× upper limit of normal) were included. Data were obtained from the routine medical documentation sheet at 0, 0.5, 1 and 2 years after diagnosis.

**Results:**

48 patients (24 patients per group) were included. Both groups did not differ in sex or age distribution, symptom burden, comorbidities, weight and BMI z-scores or routine laboratory parameters at baseline. Decline of anti-TG2-IgA-antibodies was similar in both groups. Serological remission was achieved in 6% (without EGD) and 16% (with EGD) at 0.5 years and 26% and 41% at 1 year after diagnosis with no significant differences between the two groups. Symptom relief, weight and BMI z-scores as well as hemoglobin, ferritin and mean corpuscular volume did not differ depending on the mode of diagnosis.

**Conclusion:**

Our data confirm that the biopsy-sparing diagnostic algorithm is associated with similar serological and clinical outcomes in pediatric CD patients. Thus, our findings support the further application and implementation of the “non-biopsy approach.”

## 1 Introduction

Celiac disease (CD) is a systemic autoimmune disease triggered by the ingestion of gliadin and related prolamines in genetically predisposed (HLA-DQ2/DQ8 genotype) individuals (1–3). It affects about 1% of Western civilizations and can manifest itself with diverse intestinal (diarrhea, abdominal bloating) and extraintestinal (failure to thrive, fatigue etc.) symptoms (4, 5). The disease is characterized by a TH-1-mediated immune response leading to an increase in intraepithelial lymphocytes, crypt hyperplasia and villous atrophy (1). These histopathological features were used for decades as the gold standard for diagnostics (6, 7). However, since the discovery of the role of transglutaminase 2 (TG2) as the main autoantigen of CD, diagnostic patterns have changed tremendously (8, 9). Standardized ELISAs (enzyme-linked immunosorbent-assay) detecting anti-TG2-IgA-antibodies have emerged as primary diagnostic tool showing a pooled sensitivity up to 91% and 98% in adult and pediatric patients (10). In addition, antibody titers show a positive correlation with the histopathological severity of intestinal mucosal damage (11, 12). Finally, high anti-TG2-IgA-levels ≥ 10× the upper limit of normal (ULN) are characterized by a high positive predictive value (>95%) for the diagnosis of CD (13–16). This fact led the European Society for Pediatric Gastroenterology, Hepatology and Nutrition (ESPGHAN) to adjust their diagnostic algorithm in 2012 (17). Since then, the so called “non-biopsy approach” is established in the diagnosis of CD in children. The applicability and validity of this algorithm was confirmed and expanded as part of the new guidelines (18). Currently, in patients presenting with anti-TG2-IgA-levels ≥ 10× ULN and positive anti-EMA-IgA-antibodies (in a second blood sample) neither endoscopic/histopathological confirmation nor HLA-typing is required, even in asymptomatic individuals (18). The last decade has proven that the diagnostic esophagogastroduodenoscopy (EGD) and histopathology can be omitted in patients fulfilling those criteria without missing other pathologies (16, 18).

Nonetheless, there are concerns with this diagnostic approach due to a potentially reduced acceptance of the diagnosis by patients and their families, especially among asymptomatic individuals. This may in turn compromise long-term adherence to the gluten-free diet (19). From the patient and family perspective, histopathological confirmation may confer greater diagnostic credibility and objectivity. To date, there is limited evidence directly comparing biopsy-based and non-biopsy diagnostic pathways in eligible patients and their long-term outcomes.

Therefore, the aim of this study was to compare the disease course of patients with anti-TG2-IgA-antibodies ≥ 10× ULN who either underwent or omitted EGD.

## 2 Materials and methods

### 2.1 Study design and patients

Retrospective data from CD patients diagnosed and followed from 2019 to 2024 were obtained at the pediatric gastroenterology departments of the university hospital in Giessen and in Marburg. Only patients with initial anti-TG2-IgA-antibodies ≥ 10× ULN were included in the analysis. Within this cohort, all patients who omitted EGD were identified. For each patient who did not perform EGD, one patient with high anti-TG2-IgA-antibodies ≥ 10× ULN who underwent EGD was matched for sex, age and comorbidities.

### 2.2 Data collection and outcome measures

Clinical data and routine laboratory parameters were collected from the standard medical documentation. Follow-up visits were assigned to rounded time intervals of 0.5, 1, and 2 years after initial diagnosis. The clinical data included intestinal symptoms (abdominal pain, bloating, diarrhea, constipation, varying stool pattern), extraintestinal symptoms (anemia, skin lesions, pruritus, fatigue, loss of appetite/picky eating behavior, impaired growth, weight loss, irritability, brittle nails, pallor and headaches), comorbidities, weight-, height- and BMI-(body mass index) z-scores. Z-scores were determined based on the publicly available data from the American National Health and Nutrition Examination Survey^1^.

Serological follow-up was conducted using the anti-TG2-IgA-antibody levels. Antibody levels were normalized to the corresponding cut-off level (<7 IU/mL Phadia fluorescence enzyme immunoassay (FEIA), <20 IU/mL Euroimmun ELISA, in-house standard) of the laboratory. Accordingly, all antibody levels exceeding ≥ 10× ULN were assigned a value of 100%. Serological remission was defined as a decline of antibody levels below 10%, corresponding to a drop below the cut-off. FEIA (3 patients without EGD, 1 patient with EGD) and ELISA tests were used for initial diagnostics, whereas all follow-up assessments were done exclusively with the ELISA assay.

The additional laboratory parameters included hemoglobin (Hb, g/L), ferritin (ng/mL), mean corpuscular volume (MCV, fL), alkaline phosphatase (AP, U/L) and thyroid stimulating hormone (TSH, mU/L). In addition, anemia (Hb < 11 g/dL), hypoferritinemia (ferritin < 12 ng/mL), and microcytosis (MCV < 75 fL) were analyzed as categorical variables.

The primary aim of the study was to compare the decline in anti-TG2-IgA-antibody levels and the rate of serological remission at each time point between patients with or without EGD. The secondary endpoints included differences between both groups in terms of symptom relief, changes in anthropometric measurements and laboratory values over the course of the disease.

For exploratory subgroup analyses, patients were stratified by sex and age (≤6 vs. >6 years). The age cut-off was selected because it approximated the median age of the cohort, resulting in similarly sized groups, and because it coincides with the typical age of school entry, representing a clinically relevant transition in dietary autonomy.

Finally, all patients with or without serological response were investigated. For this purpose, we analyzed all patients with persistent anti-TG2-IgA-antibodies ≥ 10× ULN at 0.5 years and/or 1 year after diagnosis (*n* = 15, termed “no serological response). Those patients were compared with children showing any serological decline (i.e., anti-TG2-IgA antibodies < 10× ULN) at these timepoints (*n* = 33, termed “early response”).

### 2.3 Statistics

Statistical analysis was performed using GraphPad Prism 10 (GraphPad Prism Software Inc., San Diego, CA, USA). Student’s unpaired two-tailed *t*-test (with Welch correction where appropriate) or Mann-Whitney U test were used where appropriate. For categorical variables, the Fisher’s exact test was used. Analysis of matched parameters was done using a mixed effects model with Dunnett’s multiple comparison test. *P*-values less than 0.05 were considered significant.

### 2.4 Ethical considerations

This study was conducted in accordance with the Declaration of Helsinki. The study was approved by the ethics committees of both medical centers (approval no. 145/24).

## 3 Results

### 3.1 Baseline characteristics of patients diagnosed with and without EGD

The complete study cohort consisted of 48 patients (24 per group) with a median age of 5.5 years (Table 1). A total of 20 patients per group (83%) were recruited from the pediatric gastroenterology department of the university hospital in Giessen. All patients undergoing EGD within the primary diagnostic algorithm were classified as Marsh III on histopathological examination.

**TABLE 1:** Patient characteristics at baseline.

	Total cohort (*n* = 48)		
	No biopsy (*n* = 24)	Biopsy (*n* = 24)	*P*
Sex			
Female	14 (58%)	14 (58%)	0.99
Male	10 (42%)	10 (42%)	
Age			
Years	5.5 (4–9.8)	5.5 (3.3–10.5)	0.8
≤6	13 (54%)	13 (54%)	0.99
>6	11 (46%)	11 (46%)	0.99
Symptoms			
Intestinal	19 (79%)	193 (79%)	0.99
Extraintestinal	13 (54%)	11 (46%)	0.79
Anti-EMA-IgA-Ab	24/24 (100% positive)	8/24 (75% positive)	0.06
HLA-typing	2/2 (HLA DQ2)	6/6 HLA DQ2	0.25
	0/0 (HLA DQ8)	1/1 HLA DQ8	
EGD during disease course	3	0	0.49
Anthropometry			
Weight z-score	−0.02 ± 0.26	0.08 ± 0.24	0.79
Height z-score	0.25 ± 0.28	0.24 ± 0.23	0.99
BMI z-score	−0.29 ± 0.36	0.15 ± 0.24	0.37
Ferritin (ng/mL)	12 (5–30)	11 (6–20)	0.7
Hb (g/L)	116 (107–127)	126 (121–133)	**0.03**
MCV (fL)	79 (72–84)	79 (77–81)	0.76
AP (U/L)	203 (154–298)	249 (186–265)	0.4
TSH (mU/L)	2.1 (1.5–2.6)	2.7 (1.8–3.5)	0.1
Comorbidities			
Iron deficiency anemia	5 (21%)	2 (8%)	0.42
Type I diabetes	0 (0%)	1 (4%)	0.99
Hypothyroidism	0 (0%)	2 (8%)	0.99
Trisomy 21	1 (4%)	2 (8%)	0.99

AP, alkaline phosphatase; EGD, esophagogastroduodenoscopy; Hb, hemoglobin; HLA, human leukocyte antigen; MCV, mean corpuscular volume; TSH, thyroid stimulating hormone.

There were no significant differences in sex or age distribution, symptom burden or comorbidities at baseline between the two groups (Table 1). Iron supplementation due to anemia was done in 4 (no EGD) and 2 patients (EGD).

Anti-EMA-IgA-antibodies were determined in 24/24 (no EGD, 100% positive) and 8/24 (EGD, 75% positive) of patients. HLA-DQ2/8 typing was performed using high-resolution PCR in a subset of patients (no EGD: 2/24 and EGD: 7/24, all positive). Baseline anthropometry was not significantly different between both groups. Baseline Hb levels were significantly lower in patients who did not undergo EGD (Table 1), while rates of anemia and microcytosis tended to be higher in this group, although these differences did not reach statistical significance (Supplementary Figure 1).

The rate of patients attending follow-up appointments at 0.5 years (no EGD: 70% vs. EGD: 88%, *p* = 0.28), 1 year (83% vs. 71%, *p* = 0.49) and 2 years (63% vs. 63%, *p* = 0.99) were similar. In patients that omitted the endoscopic diagnosis initially, three EGDs (in two patients) were performed during the disease course (on gluten-free diet) due to ongoing intestinal symptoms. No repeated EGDs were done in patients undergoing endoscopy for primary diagnosis (Table 1).

### 3.2 Decline of anti-TG2-IgA-antibodies after diagnosis

The primary aim was to compare the decline of anti-TG2-IgA-antibodies and serological remission rates as surrogates of mucosal healing and dietary adherence between both groups.

**FIGURE 1:**
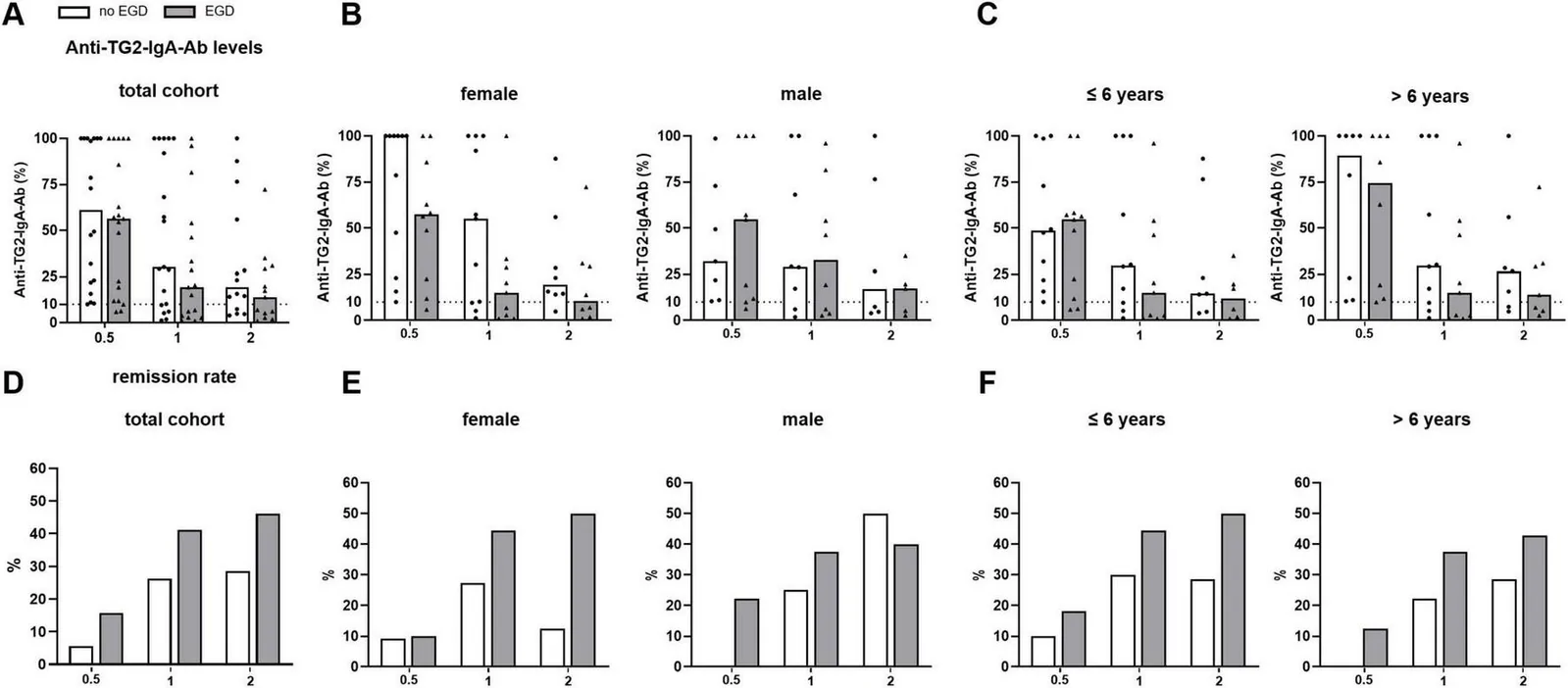
Decline of anti-TG2-IgA-antibodies and serological remission rates. Relative anti-TG2-IgA-antibody levels in the total cohort **(A)**, female and male patients **(B)** and patients ≤ 6 and >6 years of age **(C)** (data is shown as median). Rate of serological remission in the total cohort **(D)**, female and male patients **(E)** and patients ≤ 6 and >6 years of age **(F)**. *X*-axis: Time in years after diagnosis.

In the total cohort of 48 patients, there was no significant difference in the decrease in antibody titers at 0.5, 1 and 2 years between patients with or without EGD (Figure 1A). Median relative anti-TG2-IgA-antibodies dropped to 61 (no EGD) and 56 (EGD) % at 0.5 years, 30% and 19% at 1 year and 19% and 14% at 2 years, respectively (Figure 1A). Next, we analyzed subgroups including female/male patients and patients ≤ 6/>6 years. Antibody titers appeared to be higher in female patients who omitted the EGD throughout the complete observation period (Figure 1B). However, there was only a borderline significant difference in antibody titers between both groups at 1 year after diagnosis [no EGD: median 55; interquartile range (IQR) 10–100 vs. EGD: 15; 2–31; *p* = 0.13]. In all other subgroups anti-TG2-IgA-antibody titers did not differ between patients with or without EGD during the disease course (Figures 1B, C).

Importantly, in male patients omitting EGD, we observed a trend toward lower antibody titers compared to female patients at 0.5 years after diagnosis [female: median 100; (IQR) 23–100 vs. male: 32; 11%–73%; *p* = 0.09]. In patients older than 6 years of age, there was a trend for higher anti-TG2-IgA-antibody titers at 0.5 years compared to children ≤ 6 years of age. However, these differences did not reach statistical significance.

We next analyzed the serological remission rates, which increased comparably throughout the observation period (no EGD: 6/EGD: 16% at 0.5 years, 26/41% at 1 year, 29/46% at 2 years) in both patient groups (Figure 1D). The pattern of constantly rising serological remission rates was observed in all subgroups (Figures 1E, F). In female patients and patients older than 6 years of age, remission rates appeared to be higher in those children who underwent EGD. However, these differences were not significant. Finally, serological remission rates did not differ between female vs. male patients and patients ≤ 6 years vs. >6 years of age.

### 3.3 Symptomatic response during the disease course

We then investigated the course of intestinal and extraintestinal symptoms in patients with high anti-TG2-IgA-antibodies with or without EGD as a surrogate of clinical response to the gluten-free diet.

**FIGURE 2:**
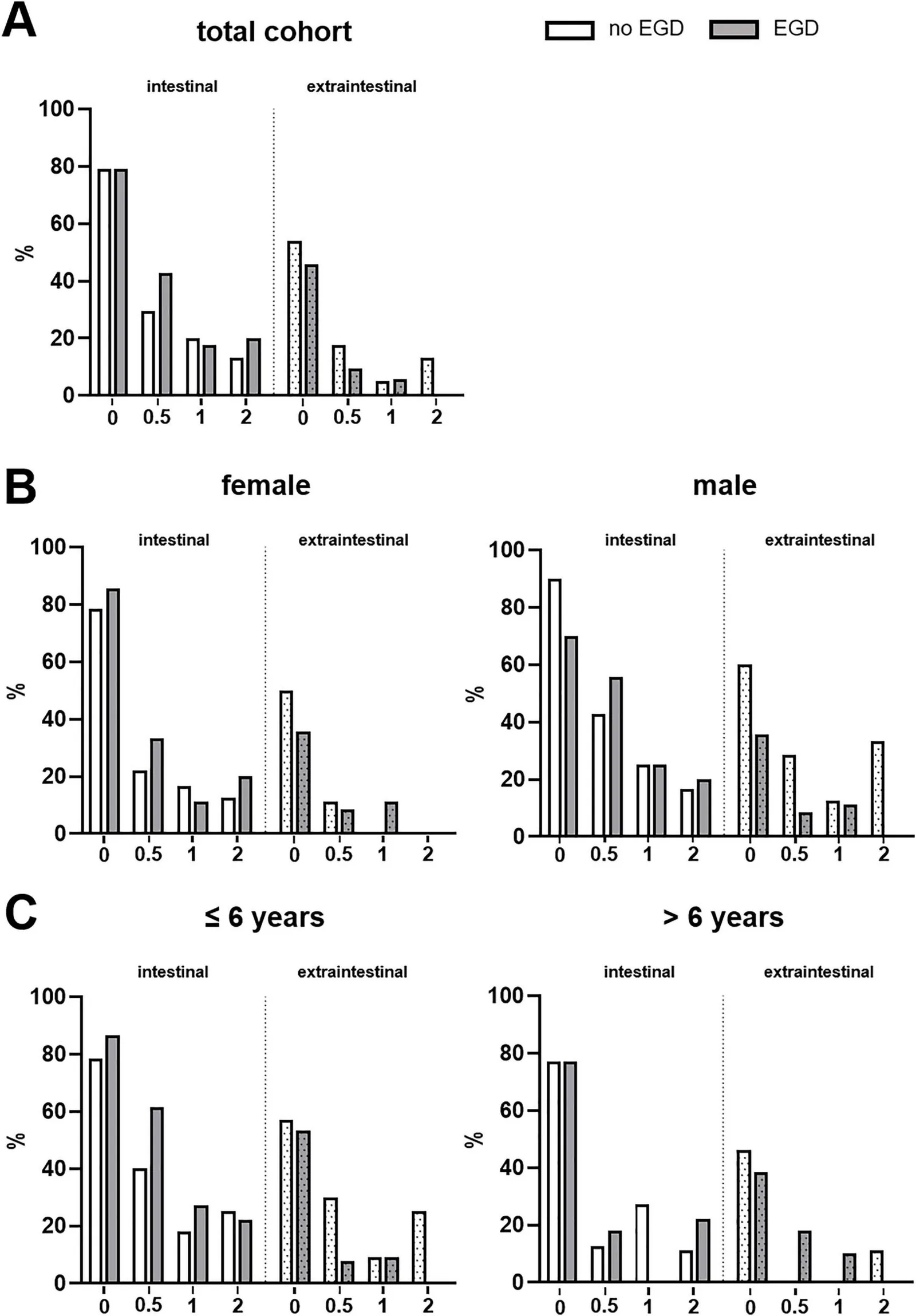
Decline of intestinal and extraintestinal symptom rates. Symptom rates in the total cohort **(A)**, female and male patients **(B)** and patients ≤ 6 and >6 years of age **(C)**. *X*-axis: Time in years after diagnosis. Sample size **(A)**; intestinal/extraintestinal symptoms: no EGD: *n* = 24 (0 years), 17 (0.5 years), 20 (1 year), 15 (2 years); EGD: *n* = 24 (0 years), 21 (0.5 years), 17 (1 year), 15 (2 years).

At baseline, the majority of patients in both groups presented with intestinal (no EGD: 79 and EGD: 79%) and/or extraintestinal (no EGD: 54 and EGD: 46%) symptoms (Figure 2A and Table 1). Only 2 patients per group (8%) did not report any symptoms. Both intestinal and extraintestinal symptoms declined rapidly within 0.5 years. At this timepoint, only 29% (no EGD) and 43% (EGD) of patients reported intestinal symptoms (Figure 2A). The percentage of patients suffering from extraintestinal symptoms declined as well (no EGD: 18%, EGD: 10%, Figure 2A). Afterwards the rate of patients with symptoms remained at low levels (<20%) at 1 and 2 years after diagnosis (Figure 2A). There were no significant differences in symptom rates between patients with or without EGD during the disease course.

In order to analyze symptom rates in further detail, we examined symptoms separately for female and male patients as well as for patients ≤ 6 and >6 years of age (Figures 2B, C). There were no significant differences in symptom burden during the disease course between patients with or without EGD in these subgroups.

However, the rate of patients presenting with symptoms appeared to be higher in male compared to female patients throughout the disease course (e.g., intestinal symptoms at 0.5 years 22 (female) vs. 43 (male) %, extraintestinal symptoms at 0.5 years 11% vs. 29%), but this did not reach statistical significance (Figure 2B). In addition, symptom burden was higher in patients ≤ 6 years of age compared to older children (Figure 2C). Here, intestinal symptoms were significantly more frequent in younger patients [EGD: intestinal symptoms at 0.5 years 62 (≤6 years) vs. 18% (>6 years), *p* < 0.05].

### 3.4 Development of anthropometric measurements during the disease course

**FIGURE 3:**
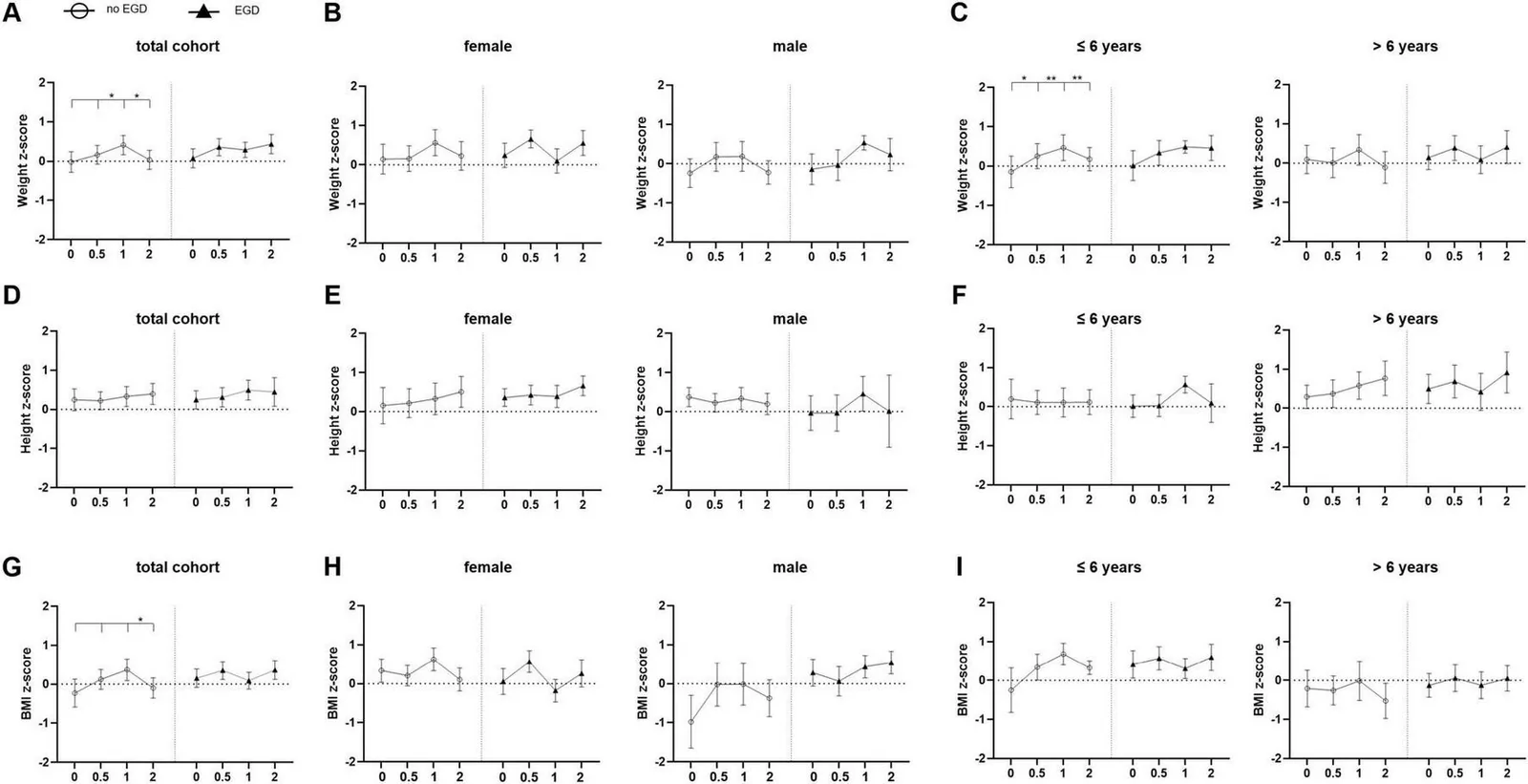
Longitudinal changes in weight, height and BMI z-scores. Mean weight z-scores of the total cohort **(A)**, female and male patients **(B)** and patients ≤ 6 and >6 years of age **(C)**. Mean height z-scores of the total cohort **(D)**, female and male patients **(E)** and patients ≤ 6 and >6 years of age **(F)**. Mean BMI z-scores of the total cohort **(G)**, female and male patients **(H)** and patients ≤ 6 and >6 years of age **(I)**. Data is shown as mean ± standard error of mean (SEM). *p < 0.05; **p < 0.01. *X*-axis: Time in years after diagnosis. Sample size **(A)**: no EGD: *n* = 21 (0 years), 21 (0.5 years), 20 (1 year), 14 (2 years); EGD: *n* = 23 (0 years), 21 (0.5 years), 16 (1 year), 14 (2 years). Sample size **(B)**: no EGD: *n* = 21 (0 years), 21 (0.5 years), 19 (1 year), 14 (2 years); EGD: *n* = 23 (0 years), 21 (0.5 years), 16 (1 year), 14 (2 years). Sample size **(C)**: no EGD: *n* = 21 (0 years), 19 (0.5 years), 20 (1 year), 14 (2 years); EGD: *n* = 23 (0 years), 21 (0.5 years), 16 (1 year), 14 (2 years).

To further analyze the disease course of CD we investigated the development of weight, height and BMI z-scores as surrogate of nutritional status in CD patients. At baseline, patients with or without EGD did not differ in terms of z-scores (Figure 3 and Table 1).

Weight z-scores increased throughout the disease course in both groups (Figure 3A). However, there was a high interindividual variation within the groups and the increment was only significant in patients omitting EGD (Figure 3A). In the total cohort and the subgroups of female vs. male patients and patients ≤ 6 vs. >6 years of age, weight z-scores did not differ between patients diagnosed with or without EGD during the observation period (Figures 3B, C). Weight z-scores increased in patients ≤ 6 years (with significant increment in patients omitting EGD). In contrast, patients > 6 years did not show improvement of weight z-scores.

Development of height z-scores did not differ throughout the disease course between patients with and without EGD in the total cohort, female vs. male patients and patients ≤ 6 vs. >6 years of age (Figures 3D–F).

Finally, the longitudinal changes of BMI z-scores were analyzed. In line with the results of weight and height z-scores, BMI z-scores increased significantly during the disease course in patients omitting EGD, whereas there was no significant increment in those patients, where endoscopic confirmation of CD was done (Figure 3G). Development of BMI z-scores during the disease course did not differ depending on whether EGD was performed or not, in the total cohort as well as in all subgroups (Figure 3H). However, in patients ≤ 6 years of age omitting EGD, there was a trend for increasing BMI z –scores (Figure 3I), which was not observed in patients > 6 years.

### 3.5 Course of laboratory values

**FIGURE 4:**
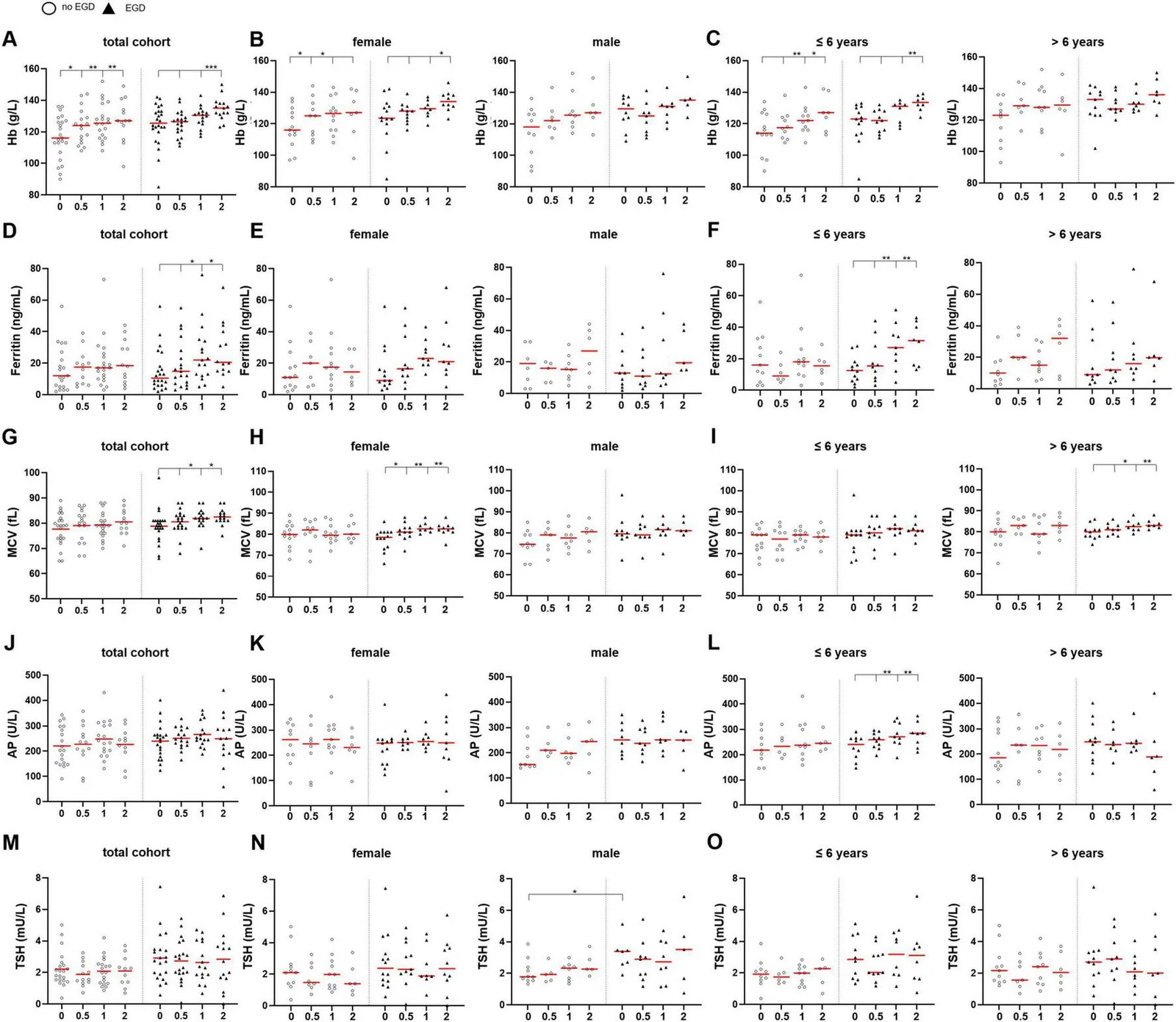
Longitudinal changes in laboratory parameters Hb, ferritin, MCV, AP and TSH during the course of the disease. Hb (g/L) levels in the total cohort **(A)**, female and male patients **(B)** and patients ≤ 6 and >6 years of age **(C)**. Ferritin (ng/mL) levels in the total cohort **(D)**, female and male patients **(E)** and patients ≤ 6 and >6 years of age **(F)**. MCV (fL) levels in the total cohort **(G)**, female and male patients **(H)** and patients ≤ 6 and >6 years of age **(I)**. AP (U/L) levels in the total cohort **(J)**, female and male patients **(K)** and patients ≤ 6 and >6 years of age **(L)**. TSH (mU/L) levels in the total cohort **(M)**, female and male patients **(N)** and patients ≤ 6 and >6 years of age **(O)**. Red line indicates median. *p < 0.05; **p < 0.01; ****p < 0.001. *X*-axis: Time in years after diagnosis.

We finally analyzed the course of routine laboratory values beginning with the hematological laboratory parameters Hb, ferritin and MCV as iron deficiency and corresponding anemia are a frequent complication of CD. Hb baseline levels were significantly lower in patients omitting EGD (Figure 4A and Table 1) but the prevalence of anemia at diagnosis did not differ between both groups (Supplementary Figure 1).

Hemoglobin levels increased over time to the same extent (Figure 4) and anemia rates improved already at 0.5 years after diagnosis irrespective whether EGD was performed or not (Supplementary Figure 1).

We observed similar patterns with significant rise of Hb levels after diagnosis in female patients and patients ≤ 6 years of age (Figures 4B, C). In those cohorts, there were no differences between patients with and without EGD. In male patients and patients older than 6 years, there was a trend toward increasing Hb levels over time, but this was not statistically significant.

We next analyzed the course of ferritin as pivotal marker of iron status. There were no significant differences in baseline ferritin levels in the total cohort as well as in the subgroups. Ferritin levels increased similarly over the course of the disease in patients with or without EGD (Figure 4D). We observed similar patterns in the subgroups (Figures 4E, F). However, the increment in ferritin levels was only significant in patients ≤ 6 years that received endoscopic confirmation (Figure 4F). There were no significant differences in ferritin levels during the course of the disease between patients with or without EGD. The rate of hypoferritinemia dropped in both groups during the course of the disease (Supplementary Figure 1). Ferritin levels below 12 ng/mL appeared to be more frequent in patients omitting EGD at 1 and 2 years after diagnosis (Supplementary Figure 1). However, this difference was not significant.

Baseline MCV levels did not differ between both groups in the total cohort as well as in the subgroups (Figures 4G–I). We observed a trend toward increasing MCV levels over time in the total cohort as well as in the subgroups. In line with this, the rate of microcytosis improved in both groups (Supplementary Figure 1). The prevalence of microcytosis appeared to be higher in patients omitting EGD at 1 and 2 years after diagnosis. However, this was not significant (Supplementary Figure 1). Overall, the course of MCV levels did not differ significantly between patients that underwent or omitted EGD in all groups (Figures 4G–I).

We next investigated the course of AP as a surrogate of bone metabolism. Baseline AP levels did not differ between patients with and without EGD in the total cohort and in the subgroups (Figures 4J–L). In patients younger than 6 years of age that underwent endoscopy, there was a significant increase of AP levels after 1 and 2 years, respectively (Figure 4L). There were no significant differences in the course of AP levels between patients with or without EGD and between the different subgroups (Figures 4J–L).

Finally, we analyzed the course of TSH as a surrogate of thyroid function. In the total cohort, TSH levels trended to be higher at baseline (*p* = 0.1, Figure 4M) and after 0.5 years (*p* = 0.05, Figure 4M) in patients that underwent EGD. In the subgroups there were significantly higher baseline TSH levels in male patients (*p* < 0.05, Figure 4N) with endoscopic confirmation. The course of TSH levels did not differ between patients who underwent or omitted EGD or in the different subgroups (Figures 4N, O).

### 3.6 Comparison between patients with or without serological response

We next sought to analyze factors associated with persistent high anti-TG2-IgA-antibodies (≥10× ULN). For this purpose, we compared all patients with anti-TG2-IgA-antibodies ≥ 10× ULN at 0.5 years and/or 1 year after diagnosis (*n* = 15, termed “no serological response”) with those showing any serological decline (*n* = 33, termed “serological response”). There were no significant differences between both groups in terms of age (serological response: median 5; IQR 3.5–9.5 vs. no serological response: 8; 4–11 years, *p* = 0.4) and sex distribution (serological response: 55 and no serological response: 67% female, *p* = 0.53). The rate of iron deficiency anemia or other comorbidities did not differ between both groups. Follow-up rates were not significantly different at 0.5 years (serological response: 79 vs. no serological response: 73%, *p* = 0.7), 1 (82% vs. 67%, *p* = 0.3) and 2 years (58% vs. 67%, *p* = 0.8) after diagnosis.

**FIGURE 5:**
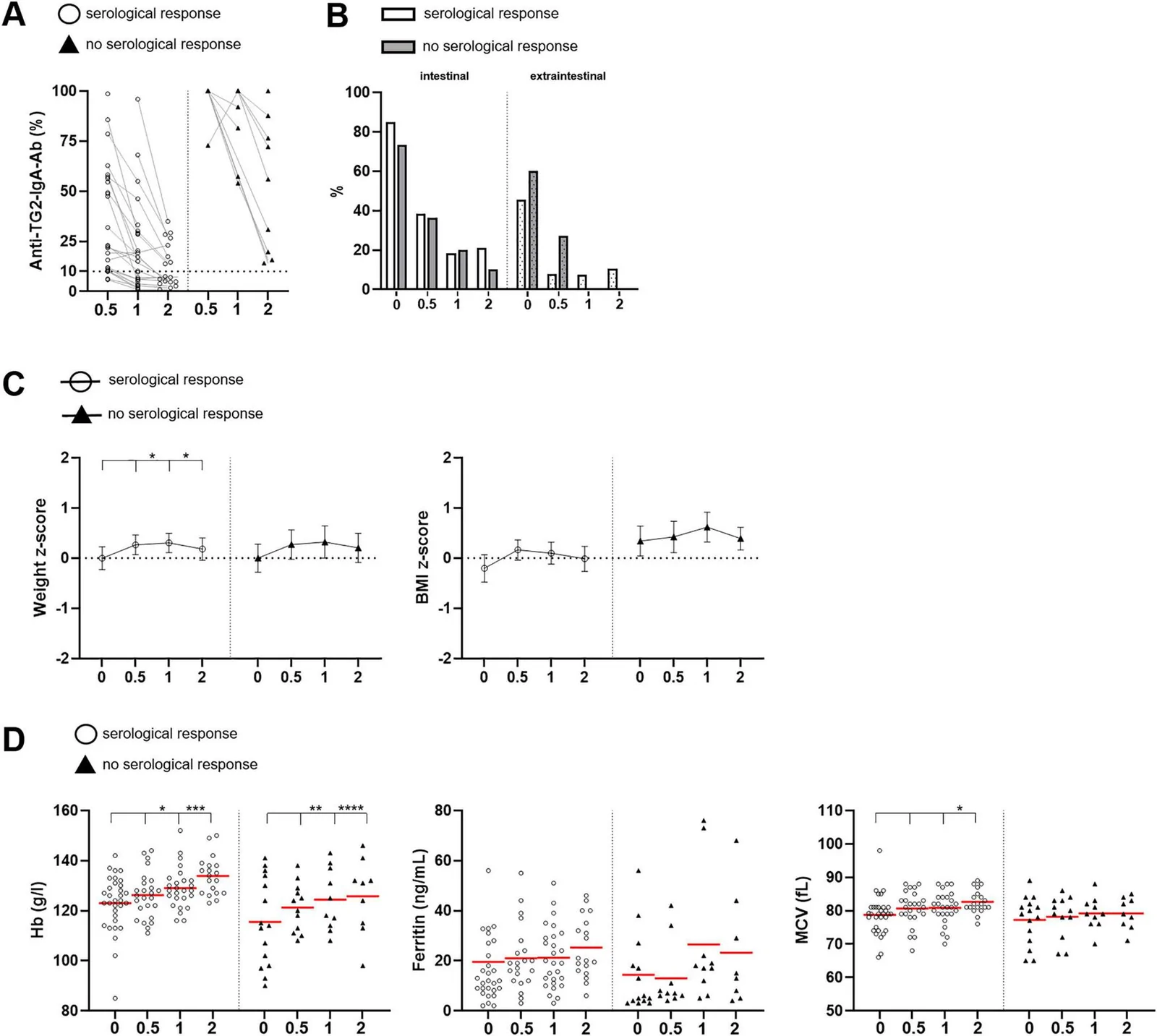
Disease outcome in patients with rapid decline in anti-TG2-IgA-antibodies (serological response) and patients with persistent high antibodies (no serological response). Individual course of anti-TG2-IgA-antibodies **(A)**. Symptom rates **(B)**. Weight- and BMI z-scores **(C)**. Laboratory values including Hb, ferritin and MCV **(D)**. Data is shown as mean ± standard error of mean (SEM). Red line indicates median. *p < 0.05; **p < 0.01. *X*-axis: Time in years after diagnosis. Samples size **(B)**: intestinal/extraintestinal symptoms: early response: *n* = 33 (0 years), 26 (0.5 years), 27 (1 year), 19 (2 years); late response: *n* = 15 (0 years), 11 (0.5 years), 10 (1 year), 10 (2 years). Sample size **(C)**: weight and BMI z-scores: early response: *n* = 30 (0 years), 328 (0.5 years), 26 (1 year), 18 (2 years); late response: *n* = 15 (0 years), 15 (0.5 years), 11 (1 year), 11 (2 years). ***p < 0.001; ****p < 0.0001.

As expected, none of the patients with persistently high anti-TG2-IgA-antibodies at 0.5 and 1 year after diagnosis achieved serological remission after 2 years (vs. 55% remission rate in patients showing serological response). In all patients of the serological response group, anti-TG2-IgA-antibody levels dropped continuously during the observation period (Figure 5A). In the majority (10/15, 67%) of patients within the no serological response group, anti-TG2-IgA-levels also dropped more than 10% during follow-up (Figure 5A). Intermittent rising of anti-TG2-IgA antibodies was only observed in one patient in the no serological response group (Figure 5A).

We next aimed to compare the clinical disease course between patients with or without serological response. Intestinal and extraintestinal symptom burden did not differ between both groups at baseline and during follow-up (Figure 5B). In addition, weight- and BMI z-scores were similar at baseline and throughout the observation period between both groups (Figure 5C).

Regarding laboratory parameters, there were no significant differences in baseline levels of Hb, ferritin and MCV between both groups (Figure 5D). However, the amount of patients with anemia was significantly higher at baseline in patients within the no serological response group (Supplementary Figure 2). In addition, the rate of hypoferritinemia was significantly higher in patients showing no serological response at 0.5 years after diagnosis (Supplementary Figure 2). However, levels of Hb, ferritin and MCV improved in both groups during the observation period.

## 4 Discussion

In this retrospective case-control study, we aimed to compare the disease course of pediatric CD patients presenting with anti-TG2-IgA-antibodies ≥ 10× ULN that underwent or omitted EGD. Baseline characteristics of both cohorts did not differ in terms of age and sex distribution, intestinal/extraintestinal symptoms and anthropometric measurements.

First, we investigated the decline in anti-TG2-IgA-antibodies and serological remission rates over time as primary outcome. There were no significant differences in median antibody titers or remission rates between both groups in the total cohort and the subgroups of female/male patients and patients ≤ 6/>6 years of age. The only significant difference was observed between female and male patients omitting EGD. Here, male patients presented with significantly lower median antibody titers at 0.5 years. This observation is in line with the results of the study by Sbravati et al. who found that female sex was associated with a longer time to normalization of CD serology (20).

In general, antibody decline and serological remission rates (no EGD/EGD: 6/16% at 0.5 years, 26/41% at 1 year, 29/46% at 2 years) of our cohort appear to be rather low compared to the existing literature, even though there is a wide range of antibody decline between the single studies. A single center retrospective study reported a remission rate of about 50% at 6–12 months (21). This finding is supported by other retrospective studies who described a normalization of anti-TG2-IgA-antibodies in 40%–50% (1 year) and 70%–80% (2 years) after diagnosis (20, 22, 23). In contrast to this, a prospective study including 228 biopsy-proven pediatric patients demonstrated lower remission rates (about 20% after 1 year) in children with high anti-TG2-IgA-antibody titers > 10× ULN (24). However, this might be also explained by the fact that the mean age of the patients was markedly older (9.5 years) than in our study, as older age is a significant predictor of delayed serological remission, probably due to lower adherence (20, 22). An additional factor that may contribute to the variability in reported serological remission rates across studies is the type of assay employed. In this context the application of the Euroimmun ELISA (in-house standard) might result in lower remission rates due to its higher sensitivity compared to the Celikey FEIA (25, 26). The comparatively low serological remission rate of our cohort may also be explained by the fact that only patients presenting with anti-TG2-IgA-antibodies ≥ 10× ULN were included, as these patients require a longer period to achieve serological normalization (24). However, selection bias cannot be excluded, as patients experiencing ongoing difficulties or concerns may have been more likely to attend follow-up visits. In addition, adherence to the gluten-free diet was not systematically assessed due to the retrospective study design.

Finally, it should be noted that serological response is only a surrogate marker of dietary adherence and mucosal healing and does not necessarily correlate with duodenal histology. In this context, retrospective studies by Leonard et al. and Stefanelli et al. demonstrated that approximately 60% of patients with persistent villous atrophy (Marsh III) at follow-up endoscopy were in serological remission (27, 28). Conversely, approximately 20%–30% of patients with histological remission (MARSH 0) still showed elevated anti-TG2-IgA-antibodies (27, 28).

To address the clinical disease course depending on the mode of diagnosis, we analyzed the rate of intestinal and extraintestinal symptoms over time. Most patients presented with intestinal (no EGD/EGD: 79/79%) and/or extraintestinal (no EGD/EGD: 54/46%) symptoms. The baseline symptom rate in our cohort is in line with the existing literature (20, 24, 29). The rate of patients with intestinal and extraintestinal symptoms already declined at 0.5 years after diagnosis. There were no significant differences in the course of CD-associated symptoms between patients with or without EGD in the total cohort, as well as in the subgroups.

We extended our analysis and investigated the development of anthropometric measurements over time. We observed increasing weight and BMI z-scores in the total cohort and especially in young patients ≤ 6 years of age with similar changes over time as found in the literature (30). Even though the increment was only significant in the group omitting EGD, there were no significant differences in weight or BMI z-score course between both groups in the complete cohort as well as in the subgroups.

Next, we examined the course of different routine laboratory parameters. First, we focused on the hematological parameters Hb, ferritin and MCV as iron deficiency anemia is a common finding in CD. For all parameters, we found continuous increments during the observation period with most prominent rise in Hb levels. In addition, the rate of anemia, hypoferritinemia and microcytosis improved rapidly irrespective of whether EGD was performed or not. Our findings are in line with existing data from Krauthammer et al. and Deora et al., who reported high rates of anemia (10%–30%) and hypoferritinemia (30%–60%) (31, 32). Other studies also reported a rapid improvement of anemia as well as of the levels of ferritin and other micronutrients within 6–12 months after diagnosis as seen in our data (32–34). In our cohort, female patients and patients ≤ 6 years showed the most significant increments in hematological parameters, especially in Hb levels and to lesser extent in ferritin and MCV levels.

Regarding AP and TSH, baseline and follow-up levels of both parameters did not differ depending on whether EGD was performed or not.

Overall, we did not observe significant differences in anti-TG2-IgA-antibodies, serological remission rates, symptomatic response, anthropometric measurements and routine laboratory values in patients with anti-TG2-IgA-antibodies ≥ 10× ULN that were diagnosed with or without EGD.

Our findings support the results of several retrospective studies from Enache et al., Klöti et al. and Kori et al., who demonstrated that the course of anti-TG2-IgA-antibodies and serological remission rates did not differ significantly depending on the diagnostic approach (35–37). This was also confirmed by a prospective study by Benelli et al. in which a similar decline in CD serology during follow-up was observed in patients diagnosed with and without EGD (38).

However, Enache et al. demonstrated a better increment in BMI z-scores and in ferritin levels in patients diagnosed with EGD (35). This contrasting finding might be explained by the fact that initial BMI z-scores (median – 1.5) were markedly lower in the study by Enache et al., whereas median ferritin levels were markedly higher compared to our cohorts (35). Enache et al. and Kori et al. also reported the follow-up rate as a surrogate of adherence. In accordance with our results there were no significantly higher rates of patients attending their follow-up visits depending on the mode of diagnosis and symptom improvement was similar in both studies (35, 36). These observations were also confirmed by the prospective study from Benelli et al. and retrospective data from Hård Af Segerstad et al. and Antonius et al., who demonstrated similar attendance and adherence rates irrespective whether biopsy was performed or not (19, 38, 39). In our work, dietary adherence was not systematically recorded, but we analyzed different surrogates of adherence such as the attendance rates, CD serology, symptom rates, anthropometric measurements and routine laboratory values.

In addition, EGD after initiation of the gluten-free diet was performed in only 2/24 and 0/24 patients who were diagnosed without or with initial endoscopy. Thus, EGD during follow-up of CD patients was a rare event.

In summary there were no significant differences in all assessed outcomes between patients who underwent or omitted endoscopic/histopathological confirmation of CD when anti-TG2-IgA-antibodies were ≥ 10× ULN.

Nonetheless, we observed significant differences between the subgroups. In this context, anti-TG2-IgA-antibodies were lower in male patients and patients ≤ 6 years of age. In addition, younger children appeared to benefit the most from the gluten-free diet, demonstrating the largest increases in weight and BMI z-scores. These findings are supported by the current literature, where an older age was associated with lower dietary adherence (40–42). The retrospective study by Dimidi et al. described that female sex was associated with lower adherence rates, which supports our finding of increased anti-TG2-IgA-antibodies in female vs. male patients (43). However, a systematic review of 13 studies by Myleus et al. found comparable adherence rates in female vs. male patients (42). In addition, the authors described multiple other factors (good knowledge about CD, membership in CD patient society, socioeconomic factors etc.) that might influence dietary adherence and which were not assessed in our study (42, 44).

Ultimately, we aimed to compare patients with and without a serological response following the diagnosis of CD. This approach was chosen because, irrespective of the diagnostic pathway, we observed both patients with persistently elevated antibody levels and those showing a marked serological response to the gluten-free diet in both groups. Overall, both patient groups did not differ in terms of sex, age distribution, comorbidities and follow-up rates. The prevalence of anemia appeared to be higher in patients showing no serological response. However, laboratory parameters as well as intestinal and extraintestinal symptoms improved similarly in both groups. Importantly, even in those patients showing a no serological response at 0.5 and 1 year after diagnosis, anti-TG2-IgA antibodies declined over time and there was only one patient in the total cohort showing an intermittent increase in antibodies in the observation period.

In general, our study is limited by its retrospective case-control design inherently carrying a risk of selection and classification bias. In addition, patients undergoing EGD despite fulfilling the criteria for the biopsy-sparing algorithm may differ in terms of disease acceptance compared to those following the non-biopsy-approach. Finally, control intervals and assessment of symptoms and dietary adherence were not standardized. In this context, prospective studies systematically evaluating dietary adherence and long-term clinical outcomes in patients diagnosed with or without EGD are warranted to further clarify the impact of the diagnostic approach.

In conclusion, this retrospective case-control study provides further evidence that the diagnosis of CD by the “non-biopsy approach” is associated with a similar outcome in terms of serological remission, symptom relief, anthropometry and routine laboratory values compared to the biopsy-based algorithm.

## Data Availability

The raw data supporting the conclusions of this article will be made available by the authors, without undue reservation.

## Footnotes

1
https://www.bcm.edu/bodycomplab/BMIapp/BMI-calculator-kids.html
